# Using a multi-method, user centred, prospective hazard analysis to assess care quality and patient safety in a care pathway

**DOI:** 10.1186/1472-6963-7-89

**Published:** 2007-06-18

**Authors:** Joanne E Dean, Allen Hutchinson, Kamisha Hamilton Escoto, Rod Lawson

**Affiliations:** 1 Section of Public Health, ScHARR, University of Sheffield, Regent Court, 30 Regent Street, Sheffield, UK, S1 4DA, UK; 2 Division of Health Policy and Management, University of Minnesota School of Public Health, Minneapolis, MN 55455, USA; 3 Department of Respiratory Medicine, University of Sheffield, Sheffield, UK

## Abstract

**Background:**

Care pathways can be complex, often involving multiple care providers and as such are recognised as containing multiple opportunities for error. Prospective hazard analysis methods may be useful for evaluating care provided across primary and secondary care pathway boundaries. These methods take into account the views of users (staff and patients) when determining where potential hazards may lie. The aim of this study is to evaluate the feasibility of prospective hazard analysis methods when assessing quality and safety in care pathways that lie across primary and secondary care boundaries.

**Methods:**

Development of a process map of the care pathway for patients entering into a Chronic Obstructive Pulmonary Disease (COPD) supported discharge programme. Triangulation of information from: care process mapping, semi-structured interviews with COPD patients, semi-structured interviews with COPD staff, two round modified Delphi study and review of prioritised quality and safety challenges by health care staff.

**Results:**

Interview themes emerged under the headings of quality of care and patient safety. Quality and safety concerns were mostly raised in relation to communication, for example, communication with other hospital teams. The three highest ranked safety concerns from the modified Delphi review were: difficulties in accessing hospital records, information transfer to primary care and failure to communicate medication changes to primary care.

**Conclusion:**

This study has demonstrated the feasibility of using mixed methods to review the quality and safety of care in a care pathway. By using multiple research methods it was possible to get a clear picture of service quality variations and also to demonstrate which points in the care pathway had real potential for patient safety incidents or system failures to occur. By using these methods to analyse one condition specific care pathway it was possible to uncover a number of hospital level problems. A number of safety challenges were systems related; these were therefore difficult to improve at care team level. Study results were used by National Health Service (NHS) stakeholders to implement solutions to problems identified in the review.

## Background

There is a substantial literature from engineering and safety critical industries on the use and methods of prospective hazard analysis [1]. A more limited literature relates to health care [2] where adapted methods such as Health Care Failure Modes and Effects Analysis [3] have been used to assess risks in high risk systems such as blood transfusion [4]. Other methods, such as socio-technical probabilistic risk analysis [5], are being used in aspects of medication safety or have been used in the study of complex environments such as anaesthesia [6].

Where they have been used in health care, most of these risk analyses have taken place in hospital-based settings, focussing on well-defined care processes. Although retrospective methods such as Root Cause Analysis are frequently used in healthcare, it is less usual to find accounts of the use of prospective hazard analysis methods across boundaries between primary and secondary care or that take account of the views of patients in determining where hazards may lie. Yet care pathways that cross the boundary between community and hospital care are recognised to contain opportunities for error, both latent and active [7].

Hence the primary purpose of this study was to investigate the feasibility and value of using mixed research methods to inform a prospective hazard analysis of risks in a care pathway that crosses primary and secondary care boundaries.

A care pathway is defined by the Department of Health for England as "*the route that a patient will take from their first contact with an NHS member of staff to the completion of their treatment" *[8].The Sheffield supported discharge programme for people with COPD was used as the study setting. The programme's aim was to reduce hospital length of stay by providing specialist, hospital-based, nursing care at home until the acute episode resolved. Therefore the care pathway for this service included care provided in hospital and care provided in the community.

Evidence suggests that early supported discharge may be a safe (using outcomes of readmission rates and mortality) and perhaps cost effective means of improving care for some people with acute exacerbations of chronic obstructive pulmonary disease [9-12]. Moreover, patients and their carers seem to prefer the option of early homecare where possible, [13] and its use is recommended in the UK National Institute for Clinical Excellence guidelines on COPD management [14].

Safety hazards in care pathways are likely to be quantified by a set of measures broader and more responsive than readmission and mortality rates [12]. For example, Reason [7] has demonstrated how safety is a dynamic process where risks change over time and where some risks are 'latent' (and difficult to foresee) while others are active and may be everyday occurrences. Dekker [15] graphically explores how departures from the routine practice envisaged in a pathway eventually become the routine. Cook [16] characterises the risks of everyday clinical practice as working within a 'safety envelope', where production pressures (such as the need to reduce length of stay to release bed space) can push clinical practice through the margin of safety into the unpredictable territory where accidents happen.

**Figure 1:**
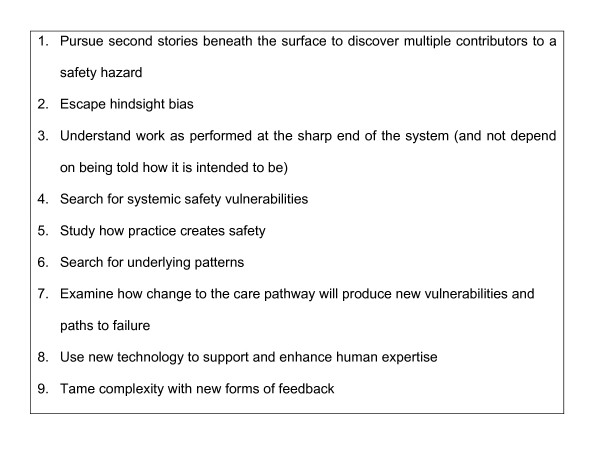
From Woods DD and Cook RI, Nine steps to move forward from error [21].

Recent reviews of quality evaluation methods [17-19] have identified a range of possible approaches for assessing quality and safety in care pathways, including methods for seeking users views and the use of Delphi methods that might be used to gain professional views on risks and potential solutions [20]. Woods and Cook [21] have drawn on the application of safety science in anaesthesia to develop a proactive check list that can be used to seek out points were safety is more vulnerable (Figure 1).

Pursuit of second stories [21] is a pre-requisite of understanding how the care pathway is actually structured, since the initial plan for any care pathway is rarely followed in every detail when put into practice. Clarity about the focus of a hazard analysis can also be gained by using standardised work process methods to establish a process map of the care pathway [22,23].

This paper reports the feasibility of using mixed methods to inform a prospective hazard analysis of the risks in a care pathway, in the context of applying the methods to a prospective hazard analysis of a COPD supported discharge care pathway.

## Methods

### Mapping the care process

Mapping of the care pathway, from admission to hospital to the point of discharge from the supported discharge programme, was undertaken iteratively through 8 one-to-one interviews and three meetings with hospital and community (Primary Care Trust) staff who were involved in designing, implementing and providing the Sheffield COPD supported discharge programme. A single observer accompanied hospital-based nursing staff during three domiciliary visits to seek further information about the supported discharge process. Finally, a joint meeting was held with hospital staff to agree the contents of the care pathway map. A standardised format [22,23] was used to create the process map. The key care decision points and processes identified from the map were subsequently the focus for the interviews with patients and staff about the process, outcome and safety of care.

Additionally, the supported discharge programme record keeping process was reviewed to determine how the information from the supported discharge scheme was retained in the hospital paper based records system.

### Review of quality and safety of care

#### Interviews

The interview schedules were based on a standardised work systems framework drawn from process engineering [24] and comprised six main themes: process understanding, work system understanding, communication, documentation, problems and suggestions for improvements and quality of care received (patient's only). All interviews were undertaken by a single researcher, who has received extensive training in qualitative research methods, including at masters level.

All staff from Sheffield Teaching Hospitals NHS Trust who were involved in the care of patients in the COPD supported discharge programme were invited to take part in the research. One staff member declined to take part in the research. Semi-structured interviews were therefore undertaken with five hospital nursing staff and two medical hospital staff involved with providing the supported discharge service.

Sixteen patients were approached for interview by nursing staff. Four supported discharge nurses each approached four patients who had been consecutively admitted to the supported discharge programme (16 patients in total). The nurses discussed the research with the patients and, if the patient agreed, their contact details were given to the project researcher. The project researcher telephoned the patients to discuss the research further and to arrange an interview if the patient wished to take part. All patients had recently been admitted to hospital with an exacerbation of symptoms of COPD, had been in the 14 day supported discharge programme and had subsequently been discharged to primary care. All contacted patients agreed to take part in the research and semi structured interviews were undertaken with all 16 patients.

### Analysis of the interview data

The analysis of the interview data was undertaken in 3 stages.

#### Stage 1

Interview data from staff and patients were analysed using FRAMEWORK, [25] an explicit, structured method of qualitative data analysis employing 5 distinct but interconnected stages in a systematic process. These stages are familiarisation; identifying a thematic framework; indexing; charting; mapping and interpretation.

For each stage in the analysis, one analyst (JD) developed a first draft of the results. The process was reviewed by a second analyst (AH) and the findings either confirmed or modified through joint discussion.

The authors firstly analysed data from staff and patient interviews separately, using a thematic analysis method to identify themes in the research e.g. communication. The themes were then compared across the patient and staff groups to assess for similarities and differences.

#### Stage 2

Information from the thematic analysis of patient and staff interview data was plotted on to the process of care map, together with information from the documentary review of record keeping. The purpose of this was to identify points in the care process where quality and safety might be variable and to assess where patients and staff felt care vulnerabilities may lie. For example, both staff and patients expressed concern about the re-admission process and, during the interviews, some patients discussed experiences they had had which did not match with the care process outlined on the care pathway. The care pathway was amended to show what happens in these cases and to demonstrate that care does not always happen according to a defined plan. The findings were reviewed by a second analyst (AH) and these were then agreed or modified in joint discussion.

#### Stage 3

The 23 sub-themes arising from the first two analyses of patient and staff interview data phases were brought together to look for similarities and differences. A final set of six main themes were developed, together with a number of specific safety vulnerabilities. These are described in the results section.

### Assessing relative safety risk

Analysis of the interview data from patients and staff highlighted seven specific areas or care pathway points where it was felt that care quality and or patient safety could potentially be compromised. The seven pathway areas were identified by compiling a list of all the quality of care and patient safety issues identified from the interviews with staff and patients, the observation of practice and care pathway mapping. By plotting the issues onto the pathway at the point of occurrence, it was possible to identify seven pathway points that warranted further investigation. A modified, two-stage, questionnaire based Delphi approach was used to obtain staff views about what worked well and what worked less well in the seven identified pathway areas.

The pathway points were:

Re-admission management; clinical organisation; communication within the COPD team; patient knowledge about the supported discharge programme; communication with the hospital bed bureau; information priorities; communication with primary care. Respondents to the Delphi questionnaire were also given the opportunity to mention any other issues that they felt were important.

In the first round, all supported discharge programme staff and all primary care staff who were members of the local Joint Care Planning Group were contacted (including: 2 General Practitioners, 2 Primary Care Chronic Disease Nurses, 1 Respiratory Nurse Specialist and COPD Primary Care Manager, 1 Community Healthcare Service Development Manager, 1 Health Improvement Manager for Chronic Disease, 1 Director of Public Health, 1 Occupational Therapist Service Manager) and the manager of the Medical Directorate of the local Acute hospital.

In a second round, all of the data sets (interviews, observations, modified Delphi questionnaire) were used to identify specific potential safety vulnerabilities within the supported discharge programme. Respondents were asked to rank each of these safety vulnerabilities in terms of risk to patients, using a visual analogue scale (0 = not a safety problem, 10 = significant safety challenge). Additionally, respondents were asked for their ideas on how each of the safety risks could be resolved or their impact reduced.

Finally, members from the COPD team and the local Joint Care Planning Group (responsible for commissioning the COPD programme from the hospital) used a Failure Mode Effect Analysis (FMEA) [3] approach to discuss possible solutions for three of the most highly ranked safety problems.

Patients were not invited to the FMEA meeting. This is because the majority of patients involved in the research were chronically ill, with some being on 24 hour oxygen whilst at home. Therefore it was not thought to be appropriate to invite them to what was anticipated to be a challenging, discussion based, meeting.

However, analysis of the interview data showed that in most cases patients and staff were in agreement with each other as to where the quality of care was good and where it was less good. The patient data was particularly useful for highlighting examples of specific problems, of which the hospital staff were already aware.

### Research governance

The study was reviewed for research governance purposes by the Sheffield Health and Social Care Research Consortium and Sheffield Teaching Hospitals Trust. Ethics review was undertaken by the North Sheffield Local Research Ethics Committee.

## Results

### Application of methods (detailed in Table 1)

**Table 1:** Application of methods

Method	Training	Details of method application	Perceived difficulties	Perceived advantages
Observation	1 days training with a human factors expert	Nursing activities were observed during 1 working day.	• Recording of events whilst observing	• Provides valuable information that would not be available through any other method
Care pathway development	None	Meetings/discussion with staff were held to develop the care pathway. Following the first meeting a first draft of the pathway was developed and discussed with the supported discharge care team at further meetings. The final version was agreed by all staff.	• Requires time commitments from busy health professionals • May require specialist software to create pathway	• Incorporates all relevant health care professionals' views and experiences of the care process
Interviews with staff and patients	5 day qualitative data collection and analysis course	Interviews were undertaken once the care pathway had been finalised. Each interview lasted approx 45 minutes to 1 hour. Analysis of this data accounted for the majority of time spent on data analysis	• Ethics approval probably required • Analysis is time consuming	• Provides richer and more detailed information than questionnaires

#### Mapping the care process

**Figure 2:**
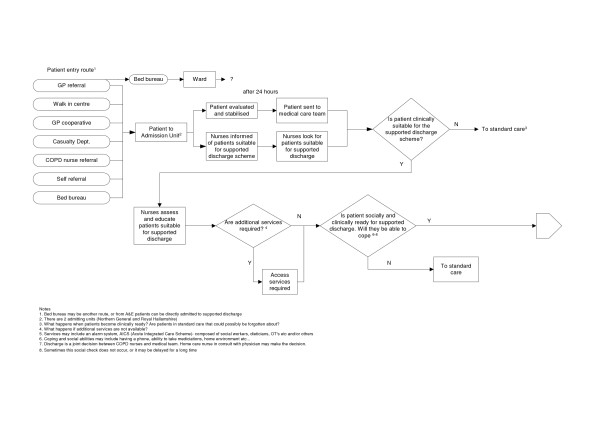
COPD Process of Care.

**Figure 3:**
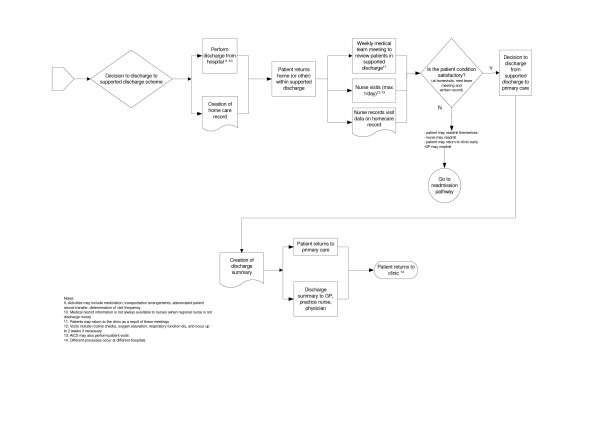
COPD Process of Care continued.

The final version of the pathway reflected a complex care process that was different between the two hospital sites within the health care Trust. Although the quality and safety of care results focus mainly on the detail of the structured discharge process, this could only be understood in the context of the whole care pathway, including admission routes and transfers between units within the hospital sites (see Figure 2 and 3).

#### Results from qualitative analysis of interview data

The six themes generated from the interviews with staff and patients can be categorised as relating primarily to patient safety or quality of care. Two interview themes lie within the quality of care category and 4 interview themes lie within the patient safety category

##### Quality of care: variation in organisation and clinical practice across hospital sites

There was provision of a differential service between the two hospital sites that provided the supported discharge service. Some COPD supported discharge services provided by one site were not provided by the other. For example, one site had a service agreement with local authority service departments to provide a home care review within 48 hours of a request. The other site did not. This variation resulted in delayed admission to the supported discharge programme for one site and, as a consequence, one site was less equipped to deliver the programme's primary aim of reducing hospital length of stay.

##### Quality of care: patient satisfaction

Patients preferred to be cared for at home rather than at hospital (14/16) and they felt more confident in returning home with the knowledge that a nurse would visit them on a daily basis. Communication between staff and patients was rated highly by most patients (14/16). One patient and family was upset over the way they perceived the supported care process had been handled in a recent short-term admission to hospital.

##### Patient safety: communication with other hospital teams

Under the supported discharge scheme, re-admission to hospital could be initiated by the respiratory nurse specialists or by the patient by telephoning the hospital bed bureau that managed the flow of hospital admissions, ensuring that patients were admitted to the relevant hospital site. This method of re-admission was in addition to traditional re-admission routes via telephoning the emergency ambulance service or the patient's General Practitioner. There was confusion about this re-admission process on various levels, particularly on the part of hospital staff who were not part of the supported discharge programme, for example in the acute admissions department, which sometimes resulted in delays in admission. On other occasions, failure by hospital teams to inform the COPD staff that a re-admission had taken place resulted in patients not being seen as quickly as intended and, in turn, extended the patient's time in a hospital bed. Patients had experience of failing to be re-admitted, having exercised their right to ask for re-admission via the hospital bed bureau. Because bed bureau telephone lines were often engaged, nursing staff sometimes had difficulty in communicating with the bureau when attempting to arrange emergency admission from the community.

##### Patient safety: communication between the chronic obstructive pulmonary disease team and primary care

For a variety of reasons, up to date information about discharged patients sometimes seemed not to reach relevant primary care staff. Nursing staff rarely managed to speak to primary care staff. The COPD staff had to make contact via the practice receptionist using the same telephone line as patients. This telephone line was often busy and on most occasions the COPD staff could only leave a message as the health professional they wished to speak to was busy. Instead, the COPD staff regularly faxed and posted documentation to the general practice. However, this did not always reach the relevant staff member. Some difficulties were structural. For instance, faxed copies of discharge summaries failed to reach primary care because, in some city general practices, fax machines were switched off on afternoons when the practices are closed.

##### Patient safety: telephone communications

Technical difficulties in telephone communications occurred regularly between staff and between staff and patients. The supported discharge programme staff spent over half of their time in the community and patients contact staff via mobile phone. But hospital rules require that mobile phones are switched off when staff are on hospital premises so the usual route for patient to staff contact was not always available.

One of the criteria for selection of patients for the supported discharge programme was that there should be an outgoing call landline in the patient's house so that a patient could call for assistance if their condition deteriorated. But in practice the community staff came across a variety of telephone difficulties, including land lines with no outgoing call facility, no landline and mobile phones which were uncharged or without credit.

##### Patient safety: access to medical records

A complex flow process for the paper-based patient records in the post-discharge period resulted in limited access to patient records when patients attend consultant/physician led hospital outpatient clinics. This was particularly a problem for one of the sites because the hospital records were stored on the other hospital site and administrative staff found it difficult to source the records for the clinic. This led to records routinely not being sourced for the clinic because of the pressures it placed on staff time. Therefore it was not unusual for outpatient appointments to be held without patient records of the prior admission.

#### Results from the Delphi study

Twelve potential safety challenges were identified from the interviews. These were confirmed in the first round of the Delphi survey, with no additions. In the second round the twelve potential safety challenges were scored on a visual analogue scale by eight NHS staff. A score of 1 denoted no safety risk whereas a score of 10 indicated a high safety risk. Safety challenges and their relative rankings are displayed in Table 2, where the mean rating for each safety challenge is displayed.

**Table 2:** Type of safety problem ranked by mean score on a visual analogue scale

**N**	**Safety problem descriptor**	**Mean score/10**
1	Routine difficulties with access to medical records in post discharge clinics leads to decisions being made without adequate background information	6.9
2	For a variety of reasons, information about discharged patients sometimes does not reach relevant primary care staff	6.8
3	Patients are at risk when medication changes during admission are not communicated to primary care	6.0
4	The service is vulnerable during periods of staff sickness, which may also affect staff morale	5.5
5	Difficulty in communicating with the bed bureau can put patients at risk	5.4
6	The provision of a differential service across the two hospitals may lead to a variation in the quality of the care provided	5.2
7	Some primary care staff appear to be unsure of the aim of the supported discharge programme, and of the care provided	5.0
8	Patients are at risk when patients do not bring their home care treatment/record with them on re-admission	5.0
9	Making and keeping hospital appointments can be a problem	4.9
10	Lack of clarity on the part of non-COPD Hospital Staff about the re-admission process leads to quality variation and admission delays, misdirection of patients and inefficiencies	4.6
11	Technical difficulties with telephone communications between staff and between staff and patients is a possible safety risk	4.5
12	Quality variation and inefficiencies occur because the COPD Supported Discharge Programme does not have a high priority, compared with other hospital services	3.8

The safety challenges which obtained the highest ratings were "Routine difficulties with access to medical records in post discharge clinics leads to decisions being made without adequate background information" and "For a variety of reasons, information about discharged patients sometimes does not reach relevant primary care staff". These two safety challenges received ratings of 6.9 and 6.8 respectively. Respondents also offered their ideas on solutions for each of the 12 safety challenges. The results from the two round Delphi questionnaire component of this quality and safety review were used to inform the FMEA meeting.

#### Failure Mode and Effect Analysis

Five senior NHS staff (out of an invited number of 12 staff) attended the final Failure Mode and Effect Analysis meeting to discuss the results, choosing to seek solutions to safety problems 1, 2 and 3 combined (because they both related to primary care teams) and 5 (safety problems outlined in Table 2). Participants did not review service problems during periods of sickness absence (problem 4) since this difficulty was thought to have been resolved.

However, pressure of time commitments on the NHS staff meant that, for various reasons, some staff were not able to commit to the effort required for a formal Failure Mode and Effect Analysis and all that could be achieved was a structured discussion of the safety challenges and recording of proposed solutions. Table 3 provides a summary of the solutions to the safety challenges that were identified by health care staff and discussed at the FMEA meeting.

**Table 3:** proposed solutions to safety challenges discussed at FMEA meeting

**Safety challenge**	**Solutions**
Routine difficulties with access to medical records in post discharge clinics leads to decisions being made without adequate background information	• 'Electronic' patient record (long term) • Patient held record – e.g. of consultants seen • Centralised record tracking system • Routine access to both sets of notes for post discharge clinics. • One set of notes rather than multiple • Notes available on IT systems • Patients to have high quality discharge summary • Cross city database to hold patient data, generate letters – access could be available in clinics and would hold more information than discharge summary
For a variety of reasons, information about discharged patients sometimes does not reach relevant primary care staff AND Patients are at risk when medication changes during admission are not communicated to primary care	• Patient held copy of discharge letter/fax • Extra copy in 'system' • Ask primary care if there are other ways that they think might work better, e.g. phone call for each individual patient with their GP/practice nurse (5–10 people per week – up to 6 calls per individual patient needed) • Direct professional phone line into practices • Respiratory directory – useful information to help contact, e.g. phone numbers, etc
Difficulty in communicating with the bed bureau can put patients at risk	• Increase number of telephone lines • Audit/monitor bed bureau response times, how easy it is to get through, etc. • Use of emergency care practitioners – send them to have a look to assess, make decisions re Fast Track Supported Discharge care • Tell Bed Bureau that patients/staff can't get through (formally? informally?) • Implement phone system logging ability etc. as per 999 systems • Implement telephone queuing system

## Discussion

The study has demonstrated the feasibility of using mixed methods to review the quality and safety of care provided through a care pathway. For much of the time the reviewed care pathway worked reasonably well and safely and on the whole the 16 interviewed patients were satisfied with the supported discharge process and the care they received.

Nevertheless the results also highlighted aspects of health care organisation where patients were potentially vulnerable to poor quality or unsafe care. Most health care systems contain latent safety risks, in which embedded organisational factors and local workplace factors can conspire to breach the defences of the system [7,26]. Since it is still uncommon for care pathways to be re-designed with specific attention to patient safety, including carrying out some form of prospective hazard analysis, it is not surprising that a number of safety vulnerabilities were found.

Some vulnerabilities were organisational and were exacerbated by the changing dynamics of the health care system, such as the recent merger of two hospitals. But such system weaknesses require a range of organisational developments to improve the service, many of which were outside the direct area of influence of the COPD team. These included achieving agreement with admission teams and the bed bureau over how the structured programme facilities for emergency admission might work. A particular problem seemed to be a failure to keep informed those who were still in medical staff in training positions (and who had short term appointments) about the admission processes of the supported discharge programme.

The challenge of accessing paper based medical records for some of the follow-up clinics, and the eventual resigned acceptance of the status quo after failed attempts to rectify the problem, is an example of what Dekker [15] refers to as 'deviations from the routine becoming the routine'. Electronic records may be the solution here but, in the short term, local workplace initiatives are required to improve the safety of care provided. These changes did not seem to be within the influence of the chronic obstructive pulmonary disease team – this is a hospital level problem.

Even telephone communications between staff and patients and patients and the hospital had unforeseen problems that were difficult to deal with. Having an outgoing call landline facility at their home was a criterion for patient selection to the supported discharge programme – some patients evidently slip through this selection criterion. Despite the fact that the communication problems were well known to the nursing staff, nothing had been done to tighten the patient selection criteria for the supported discharge programme.

The types of safety problems identified in Table 2 demonstrate the complexity of patient safety in care pathways that cross through primary and secondary care lines. Some of the problems are patient driven, for example when patients fail to attend appointments or do not bring their homecare record with them when they are re-admitted to hospital. Other problems are staff driven, for example, when information does not reach primary care and others are organisational issues, such as when hospital staff are unclear what the aim of the supported discharge programme is. Addressing these vulnerabilities is complex as their origins are within different entities with different motivations. However, since staff have been through the process of reviewing care quality and patient safety in the COPD supported discharge care pathway, a number of changes have been introduced as a response to the research. These include centralising the service at one hospital site and updating documentation processes.

There are methodological limitations to this study. It was undertaken in two sites (within one hospital Trust) so the findings may not be generalisable. However, the feasibility of methods may well be, even though it was sometimes difficult to keep all parties engaged in the research throughout the study period. It had been intended to undertake a prospective Health Care Failure Mode and Effect Analysis [3] as the final stage but this was not possible as a number of staff were unable to attend the meeting. The nature of health care meant that some contributors had to cancel on the day of the meeting.

## Conclusion

By triangulating information from a detailed mapping of the care pathway, from views and from concerns of users, and by ranking problems by potential severity of impact on care quality and patient safety, it was possible for health care staff to get a clear picture of service quality variations in the supported discharge programme. It was also possible to demonstrate which points in the care pathway had real potential for patient safety incidents or system failures to occur. Most of the variations in quality of care and care organisation resulted from system deficits and some required coordinated effort to achieve improvements. This analysis could also serve as a catalyst for identifying sub-processes in the supported discharge programme that are in serious need of redesign. For example, our methodologies showed that patient readmission to the hospital and transfer of patient information between the COPD team and primary care are areas that may need to be re-examined using novel design perspectives [27].

Safety can be defined in more ways than by mortality and admission rates alone and this study demonstrates how much of the potential for safety incidents at the individual level is embedded in the design and the actuality of the care pathway and its processes. Taking into account the reality of finding time in the lives of busy healthcare professionals, whose first response is to immediate healthcare pressures, the use of other methods of gathering information about prospective hazards, such as interviews of patients and staff and the use of Delphi methods to capture additional data, may be a more successful alternative to a full-scale, formal prospective hazard analysis.

## Competing interests

RL is one of the clinicians providing the supported discharge service.

## Authors' contributions

AH conceived of the study, obtained funding, oversaw the running of the study, analysed data and contributed to the development of the care pathway and the writing of the manuscript. JED obtained ethics approval, developed data collection materials conducted fieldwork, analysed data and contributed to the development of the care pathway and the writing of the manuscript. KHE contributed to the literature searches, development of the care pathway, development of interview schedules and the writing of the manuscript. RL was involved in the conception of the project, project meetings, data collection and contributed to the development of the care pathway and the writing of the manuscript. All authors read and approved the final manuscript.

## Pre-publication history

The pre-publication history for this paper can be accessed here:


